# Automated predictive analytics tool for rainfall forecasting

**DOI:** 10.1038/s41598-021-95735-8

**Published:** 2021-09-06

**Authors:** Maulin Raval, Pavithra Sivashanmugam, Vu Pham, Hardik Gohel, Ajeet Kaushik, Yun Wan

**Affiliations:** 1 Applied Artificial Intelligence Laboratory, University of Houston-Victoria, Victoria, USA; 2 NanoBioTech Laboratory Florida Polytechnic University, Lakeland, USA

**Keywords:** Environmental sciences, Engineering, Mathematics and computing

## Abstract

Australia faces a dryness disaster whose impact may be mitigated by rainfall prediction. Being an incredibly challenging task, yet accurate prediction of rainfall plays an enormous role in policy making, decision making and organizing sustainable water resource systems. The ability to accurately predict rainfall patterns empowers civilizations. Though short-term rainfall predictions are provided by meteorological systems, long-term prediction of rainfall is challenging and has a lot of factors that lead to uncertainty. Historically, various researchers have experimented with several machine learning techniques in rainfall prediction with given weather conditions. However, in places like Australia where the climate is variable, finding the best method to model the complex rainfall process is a major challenge. The aim of this paper is to: (a) predict rainfall using machine learning algorithms and comparing the performance of different models. (b) Develop an optimized neural network and develop a prediction model using the neural network (c) to do a comparative study of new and existing prediction techniques using Australian rainfall data. In this paper, rainfall data collected over a span of ten years from 2007 to 2017, with the input from 26 geographically diverse locations have been used to develop the predictive models. The data was divided into training and testing sets for validation purposes. The results show that both traditional and neural network-based machine learning models can predict rainfall with more precision.

## Introduction

Water is crucial and essential for sustaining life on earth. Water plays a key role in the development of the economic, social and environment of a region. Every aspect of life, be it life’s survival, agriculture, industries, livestock everything depends on the availability of water. Increase in population, urbanization, demand for expanded agriculture, modernized living standards have increased the demand for water^1^. Water is a renewable resource, and it is transferred between the ocean, atmosphere, and the land (through rainfall)^2^. Rainfall is a life-sustaining water resource, and its variability influences the water availability across any region. Rain also irrigates all flora and fauna. When water is added to rivers and dams in turn, it may be used to generate electricity through hydropower.

Australia is the driest inhabited continent with 70% of the continent classified as desert or semi-desert. This island continent depends on rainfall for its water supply^3,4^. Just like any other region, variation in rainfall often influences water availability across Australia. The continent encounters varied rainfall patterns including dryness (absence of rainfall), floods (excessive rainfall) and droughts^5^. Some examples are the Millenium drought, which lasted over a decade from 1995 to 2009^6^, the 1970s dry shift in southwest Australia^7^, and the widespread flooding from 2009 to 2012 in the eastern Australian regions^8^. It has the highest rainfall in the tropical regions in the north and dry and deserted regions in the interior. These changes in the rainfall creates serious issues in water availability, management, and future resource planning.

Researchers have developed many algorithms to improve accuracy of rainfall predictions. Predicting rainfall accurately is a complex process, which needs improvement continuously. Our rainfall prediction approach lies within the traditional synoptic weather prediction that involves collecting and analyzing large data, while we will use and compare various data science techniques for classification, model selection, sampling techniques etc. to train and test our models. In this paper, different machine learning models are evaluated and compared their performances with each other. We have attempted to develop an optimized neural network-based machine learning model to predict rainfall. The models use GridSearchCV to find the best parameters for different models.

## Literature survey

Water is essential to all livelihood and all civil and industrial applications. Accurate rainfall prediction is important for planning and scheduling of these activities^9^. There is numerous literature available on different rainfall prediction approaches including but not limited to data mining, artificial neural networks and machine learning^10^.

Hu^11^ was one of the key people who started using data science and artificial neural network techniques in weather forecasting. He used Adaline, which is an adaptive system for classifying patterns, which was trained at sea-level atmospheric pressures and wind direction changes over a span of 24 h. Adaline was able to make “rain vs. no-rain” forecasts for the San Francisco area on over ninety independent cases. The predictions were compared with actual United States Weather Bureau forecasts and the results were favorable. Hu’s work was foundational in developing advanced and accurate rainfall techniques. Cook^12^ presented a data science technique to predict average air temperatures. Among many algorithms they had tested, back-propagation learning algorithm was one of them.

An important research work in data-science-based rainfall forecasting was undertaken by French^13^ with a team of researchers, who employed a neural network model to forecast two-class rainfall predictions 1 h in advance. Michaelides^14^ and the team have compared performance of a neural network model with multiple linear regressions in extrapolating and simulating missing rainfall data over Cyprus.

Data mining techniques are also extremely popular in weather predictions. Chauhan and Thakur^15^ broadly define various weather prediction techniques into three broad categories:

1. Synoptic weather prediction: A traditional approach in weather prediction and refers to observing the feature weather elements within a specific time of observations at a consistent frequency. It involves collecting data daily and analyzing the enormous collection of observed data to find the patterns of evidence.
2. Numerical weather prediction: Uses computer analytical power to do weather prediction and allows the computer program to build models rather than human-defined parametric modeling after visualizing the observed data. This is often combined with artificial intelligence methods.
3. Statistical weather prediction: Often coupled with numerical weather prediction methods and uses the main underlying assumption as “the future weather patterns will be a repetition of the past weather patterns”.

Petre^16^ uses a decision tree and CART algorithm for rainfall prediction using the recorded data between 2002 and 2005. Sharif and team^17^ have used a clustering method with K-nearest neighbors to find the underlying patterns in a large weather dataset. They achieved high prediction accuracy of rainfall, temperatures, and humidity.

Our main goal is to develop a model that learns rainfall patterns and predicts whether it will rain the next day.

## Data source

**Figure 1:**
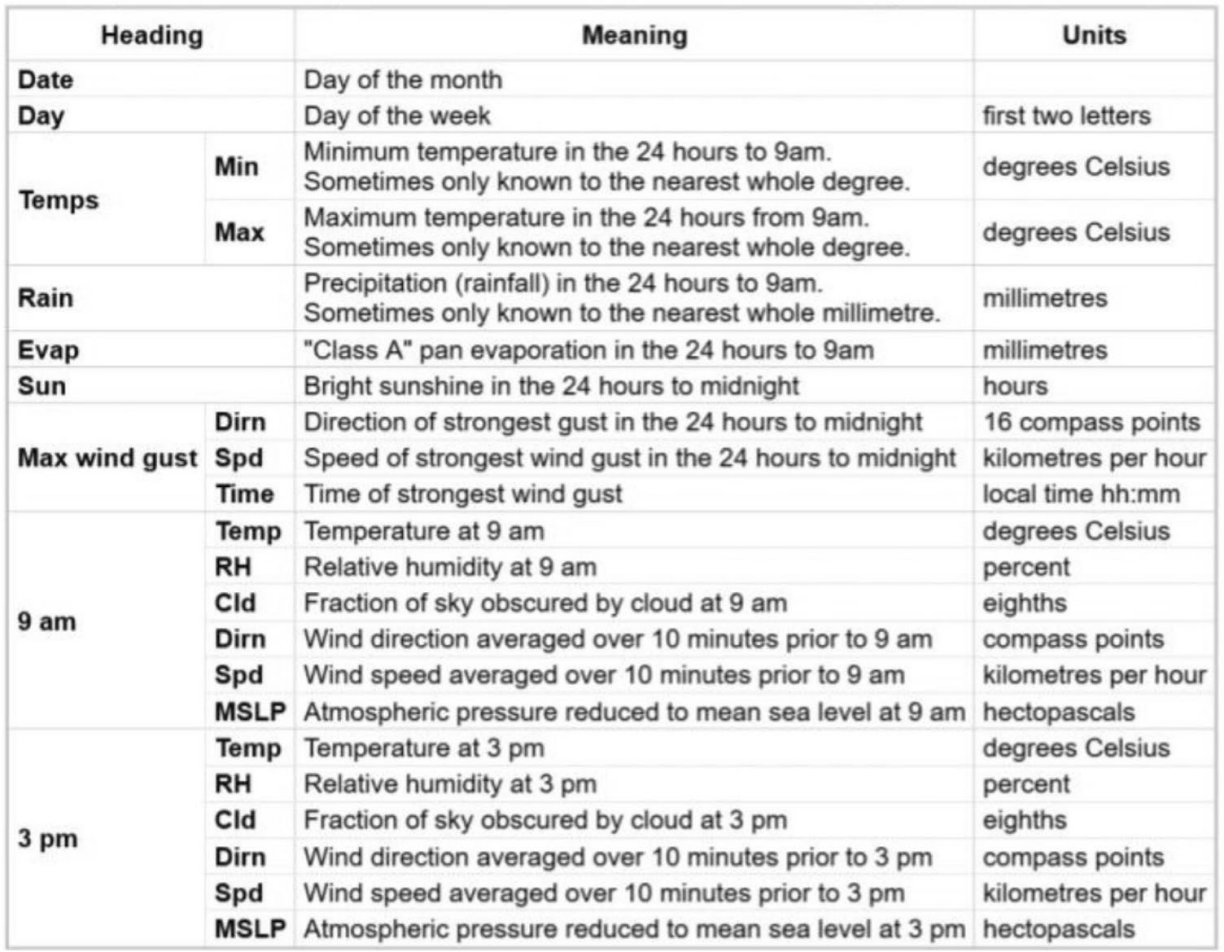
Data parameters.

We used the dataset containing 10 years’ worth of daily weather observations from multiple Australian weather stations (climate data online, Bureau of meteorology, Australian government)^18^. We use a total of 142,194 sets of observations to test, train and compare our prediction models. These observations are daily weather observations made at 9 am and 3 pm over a span of 10 years, from 10/31/2007 to 06/24/2017. This data is used in building various regression and classification models in this paper, including but not limited to the binary classification model on the response Rain Tomorrow. Figure 1 lists all data parameters collected.

### Data wrangling and exploratory data analysis (EDA)

We primarily use R-studio in coding and visualization of this project. We used several R libraries in our analysis. In our data, there are a total of twenty-four columns. Out of a total of 142,194 rows, there are multiple rows in the data that are missing one or more feature values. First, we perform data cleaning using dplyr library to convert the data frame to appropriate data types.

In performing data wrangling, we convert several variables like temperatures and pressures from character type to numeric type. We also convert qualitative variables like wind-direction, RainTomorrow from character type to factor type. Moreover, we convert wind speed, and number of clouds from character type to integer type. The next step is assigning ‘1’ is RainTomorrow is Yes, and ‘0’ if RainTomorrow is No.

Also, we convert real numbers rounded to two decimal places. The next step is to remove the observations with multiple missing values. Thus, after all the cleaning up, the dataset is pruned down to a total of 56,466 set of observations to work with.

Note that a data frame of 56,466 sets observation is usually quite large to work with and adds to computational time. Therefore, we use K-fold cross-validation approach to create a K-fold partition of n number of datasets and for each k experiment, use k − 1 folds for training and the held-out fold for testing. This does not have to be performed necessarily in k − 1/1 partition for training/testing but may also be compared with other combinations like k − 2/2, k − 3/3 and so one for training/held-out testing folds, according to Wei and Chen^19^. For the starter, we split the data in ten folds, using nine for training and one for testing.

It is evident from the plots that the temperature, pressure, and humidity variables are internally correlated to their morning and afternoon values. However, it is also evident that temperature and humidity demonstrate a convex relationship but are not significantly correlated. Moreover, sunshine and temperature also show a visible pattern and so does pressure and temperature, but do not have much correlation as can be confirmed from the correlation heat map. As expected, morning and afternoon features are internally correlated. Also, observe that evaporation has a correlation of 0.7 to daily maximum temperature. Further, we can also plot the response of RainTomorrow along with temperature, evaporation, humidity, and pressure^20^. The scatter plots display how the response is classified to the predictors, and boxplots displays the statistical values of the feature, at which the response is Yes or No.

**Figure 2:**
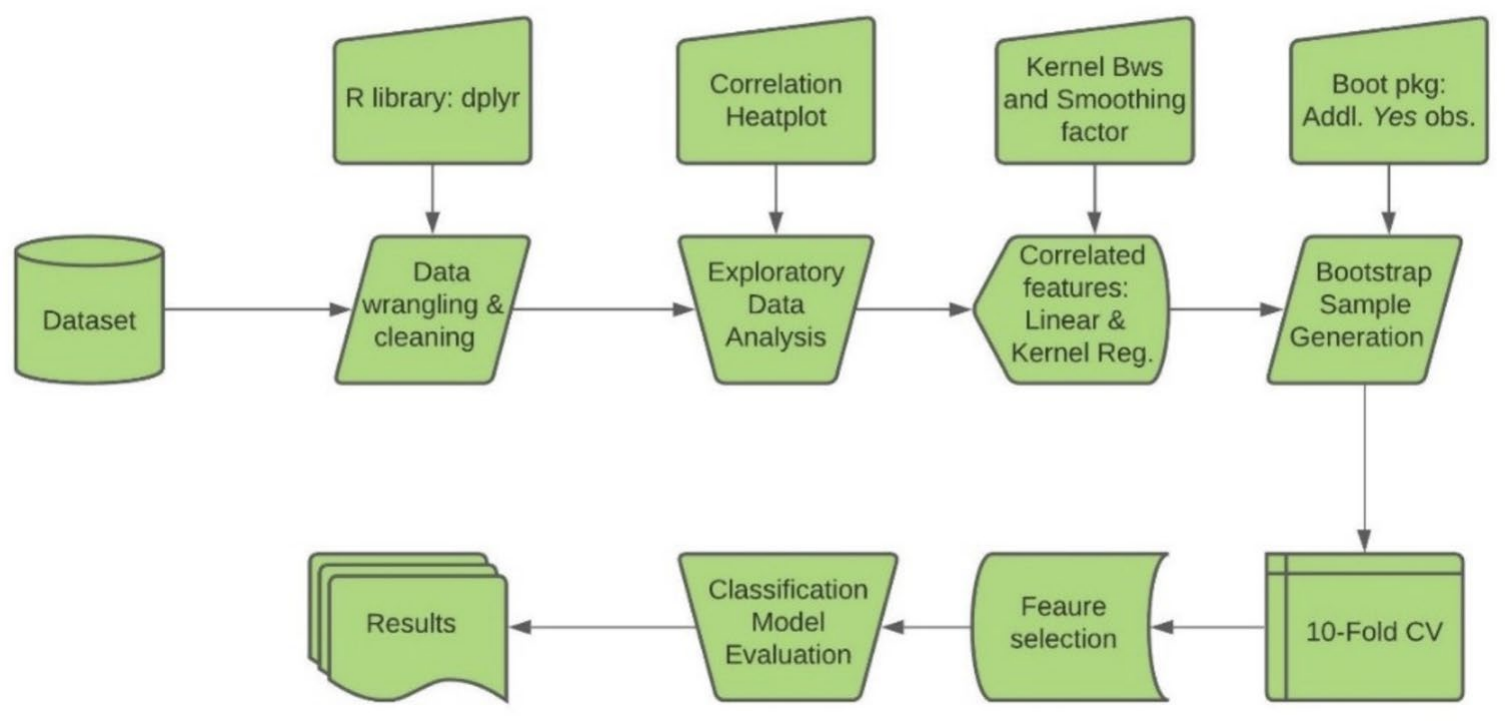
Process flow chart.

**Figure 3:**
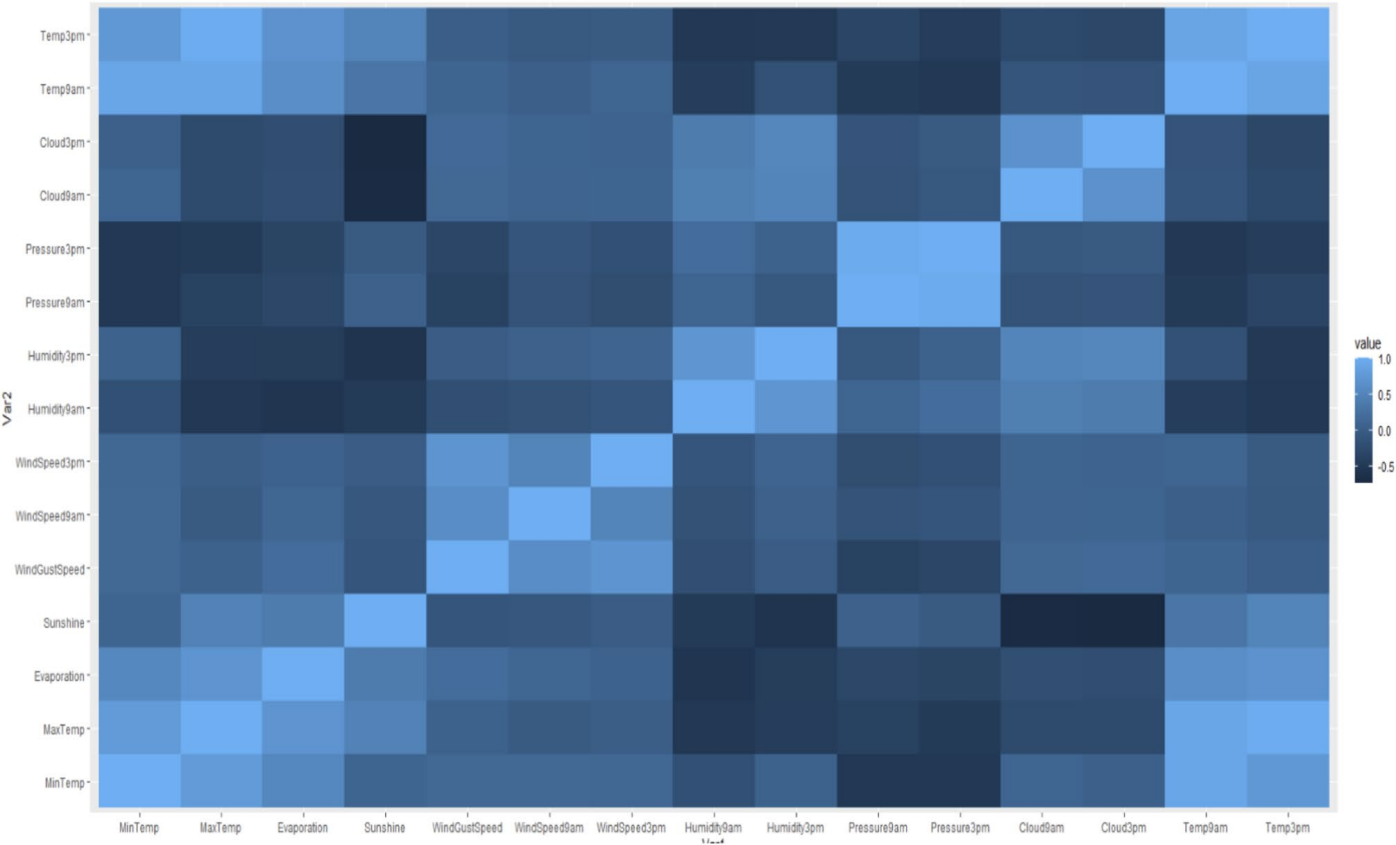
Correlation heat plot.

**Figure 4:**
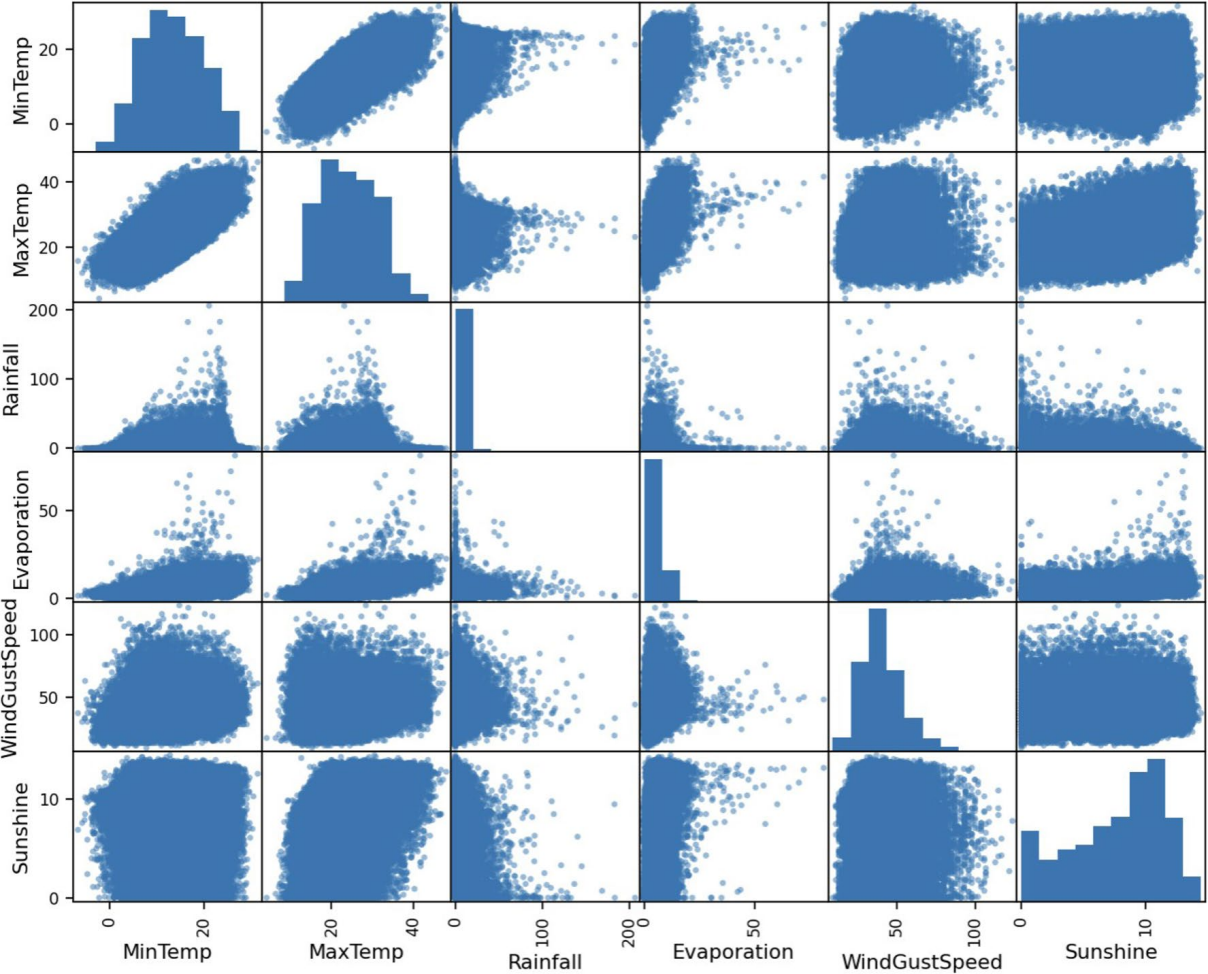
Feature scatter plot.

Figure 2 displays the process flow chart of our analysis. We first performed data wrangling and exploratory data analysis to determine significant feature correlations and relationships as shown in Figs. 3 and 4.

**Figure 5:**
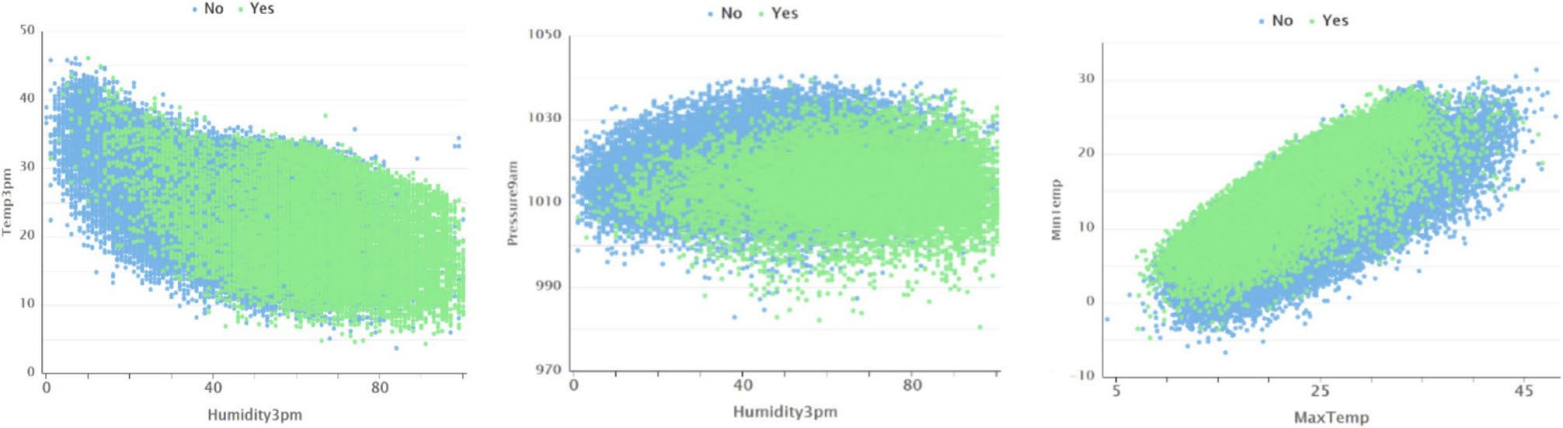
Box plots between significant features.

**Figure 6:**
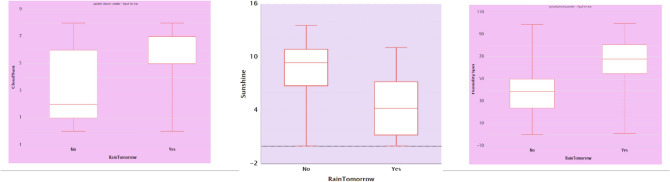
Box plots between significant features.

It is evident from scatter plots in Fig. 5 that rainfall depends on the values of temperature, humidity, pressure, and sunshine levels. For the variable RainTomorrow to have a higher probability for a Yes value, there is a minimum relative humidity level of 45%, atmospheric pressure range of 1005 and 1028 hectopascals, and lower sunshine level as evident from the boxplot (Fig. 6).

Now we have a general idea of how the data look like; after general EDA, we may explore the inter-relationships between the feature temperature, pressure and humidity using generalized logistic regression models.

### Feature selection

We explore the relationships and generate generalized linear regression models between temperature, humidity, sunshine, pressure, and evaporation. The purpose of using generalized linear regression to explore the relationship between these features is to one, see how these features depend on each other including their correlation with each other, and two, to understand which features are statistically significant^21^.

**Figure 7:**
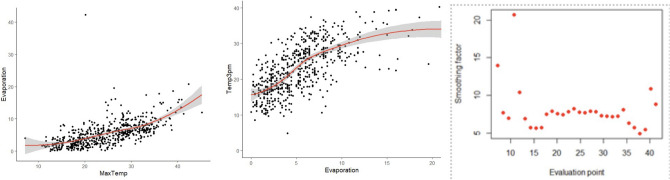
Boxplots between significant features.

**Figure 8:**
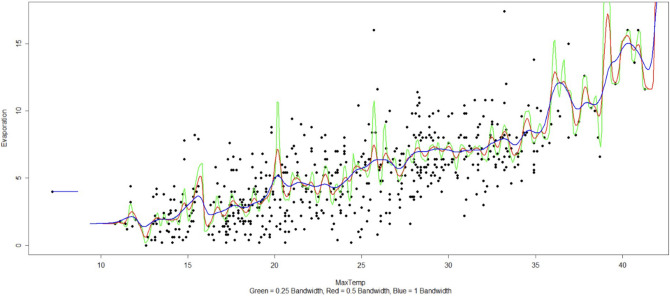
Boxplots between significant features.

For example, Fig. 7 shows that there is a quadratic trend between temperature and evaporation. Also, we determined optimal kernel bandwidth to fit a kernel regression function and observed that a kernel regression with bandwidth of 1 is a superior fit than a generalized quadratic fit. Fig. 8 presents kernel regression with three bandwidths over evaporation-temperature curve.

We use generalized linear regression to establish the relationships between correlated features. However, the outliers are affecting the model performance. So, after removing those outliers, we reproduce a kernel regression model with different bandwidths and pick an optimum bandwidth of 1. We also use bias-variance decomposition to verify the optimal kernel bandwidth and smoother^22^.

We have used the “nprobust” package of R in evaluating the kernels and selecting the right bandwidth and smoothing parameter to fit the relationship between quantitative parameters. We have used the cubic polynomial fit with Gaussian kernel to fit the relationship between Evaporation and daily MaxTemp.

After fitting the relationships between inter-dependent quantitative variables, the next step is to fit a classification model to accurately predict Yes or No response for RainTomorrow variables based on the given quantitative and qualitative features. For this, we start determining which features have a statistically significant relationship with the response. We also perform Pearson’s chi squared test with simulated p-value based on 2000 replicates to support our hypothesis^23–25^.

After running the above replications on ten-fold training and test data, we realized that statistically significant features for rainfall prediction are the fraction of sky obscured by clouds at 9 a.m., humidity and evaporation levels, sunshine, precipitation, and daily maximum temperatures. Moreover, after cleaning the data of all the NA/NaN values, we had a total of 56,421 data sets with 43,994 No values and 12,427 Yes values. We used this data which is a good sample to perform multiple cross validation experiments to evaluate and propose the high-performing models representing the population^3,26^.

### Performance

Scalability and autonomy drive performance up by allowing to promptly add more processing power, storage capacity, or network bandwidth to any network point where there is a spike of user requests. Moreover, autonomy also allows local developers and administrators freely work on their nodes to a great extent without compromising the whole connected system, therefore software can be upgraded without waiting for “approval” from other systems. Load balancing over multiple nodes connected by high-speed communication lines helps distributing heavy loads to lighter-load nodes to improve transaction operation performance.

## Model selection

For the classification problem of predicting rainfall, we compare the following models in our pursuit:

- Logistic regression
- Linear discriminant analysis
- Quadratic discriminant analysis
- K-Nearest Neighbor for classification
- Decision tree and gradient boosted trees
- Random forest
- Bernoulli Naïve Bayes
- Deep learning

**Figure 9:**
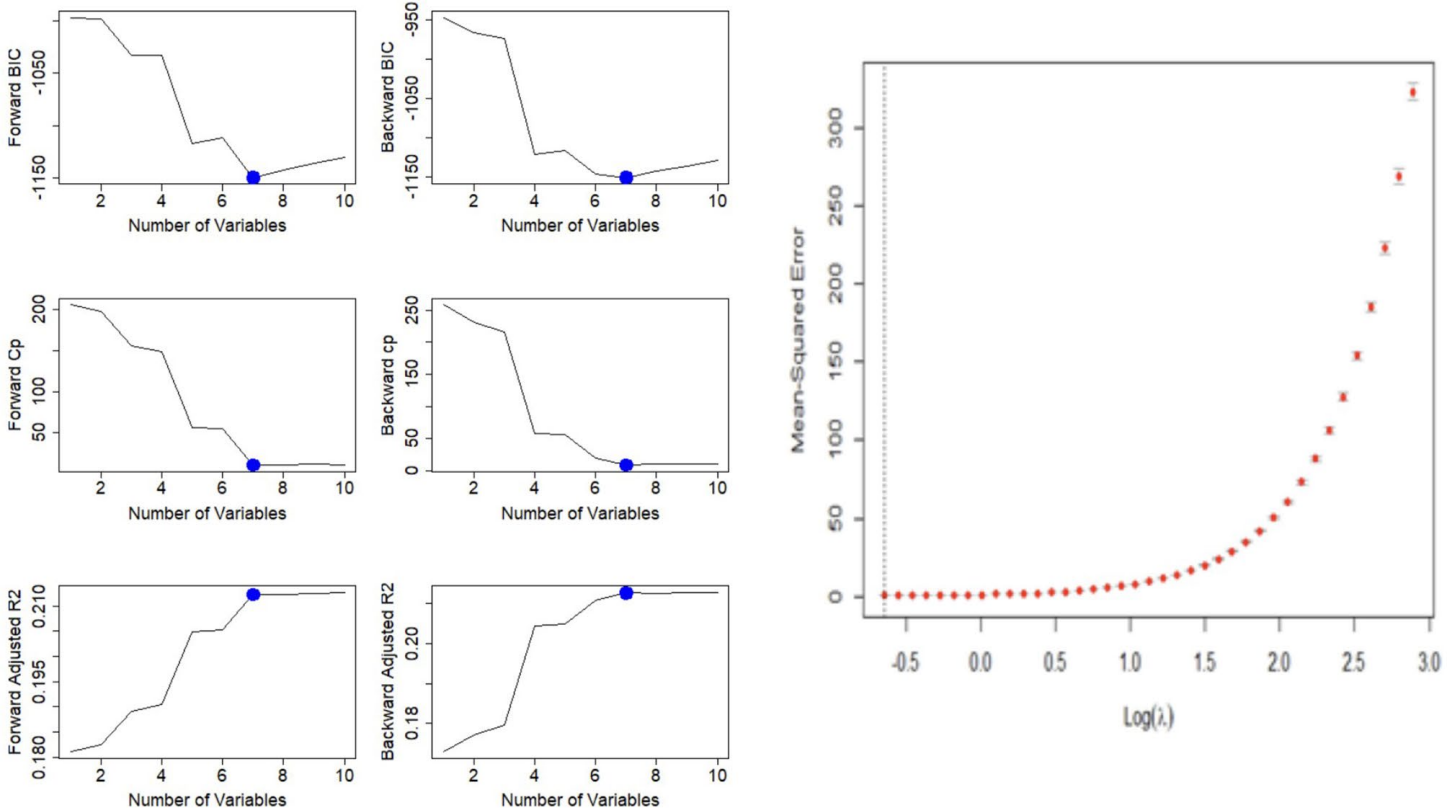
Boxplots between significant features.

To maximize true positives and minimize false positives, we optimize all models with the metric precision and f1-score. Thus, the model with the highest precision and f1-score will be considered the best. As shown in Fig. 9, we perform subset selection and find optimal subset to minimize BIC and Cp and maximize adjusted.

### Logistic regression

We performed feature engineering and logistic regression to perform predictive classification modelling. During the testing and evaluation of all the classification models, we evaluated over 500 feature set combinations and used the following set of features for logistic regression based on their statistical significance, model performance and prediction error^27^. After performing above feature engineering, we determine the following weights as the optimal weights to each of the above features with their respective coefficients for the best model performance^28^. The confusion matrix obtained (not included as part of the results) is one of the 10 different testing samples in a ten-fold cross validation test-samples.

**Figure 10:**
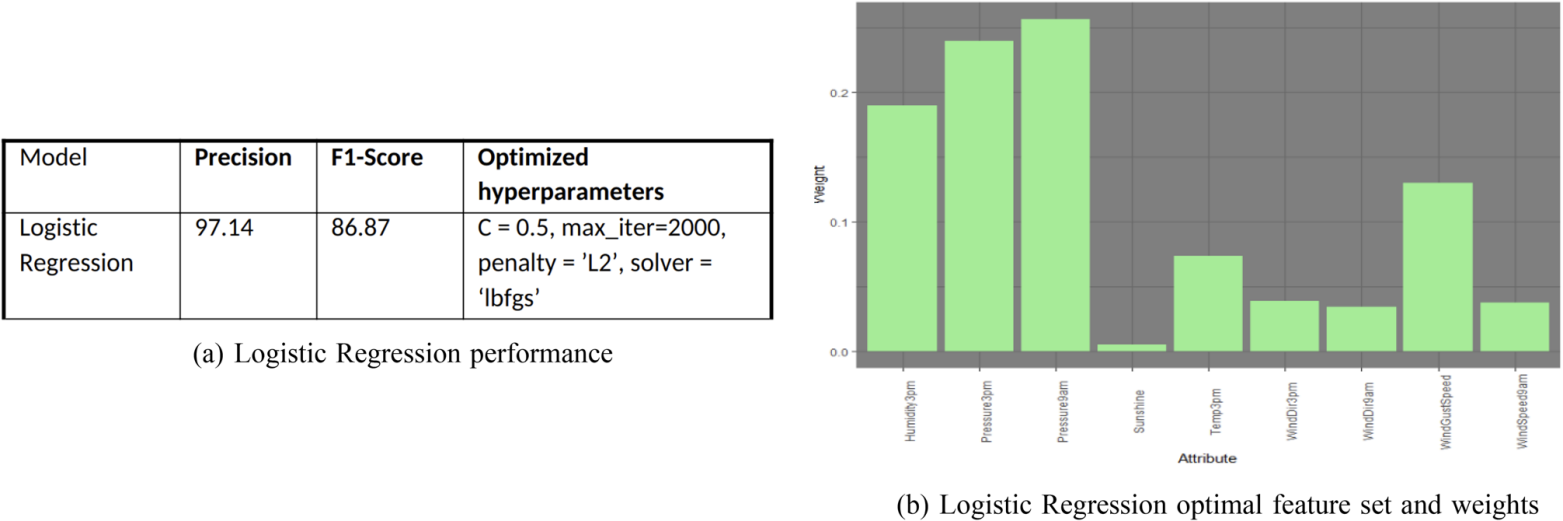
Logistic regression performance and feature set.

Figure 10a displays class precision and f1-score along with optimized hyper parameters used in the model. Figure 10b presents significant feature set and their weights in rainfall prediction. The results of gridSearchCV function is used to determine the best hyper parameters for the model.

### Linear discriminant analysis

**Figure 11:**
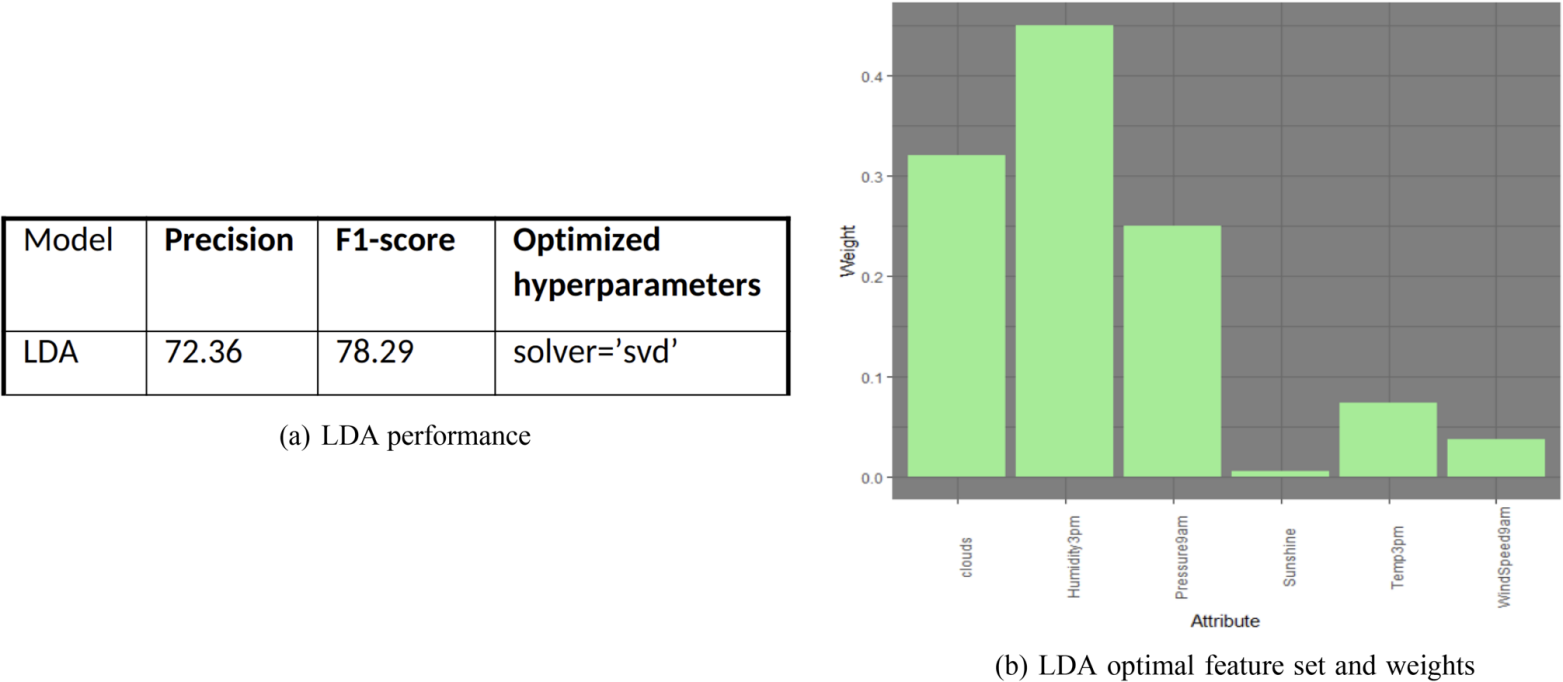
LDA performance and feature set.

We performed a similar feature engineering, model evaluation and selection just like the above, on a linear discriminant analysis classification model, and the model selected the following features for generation. Figure 11a,b show this model’s performance and its feature weights with their respective coefficients.

### Quadratic discriminant analysis

**Figure 12:**
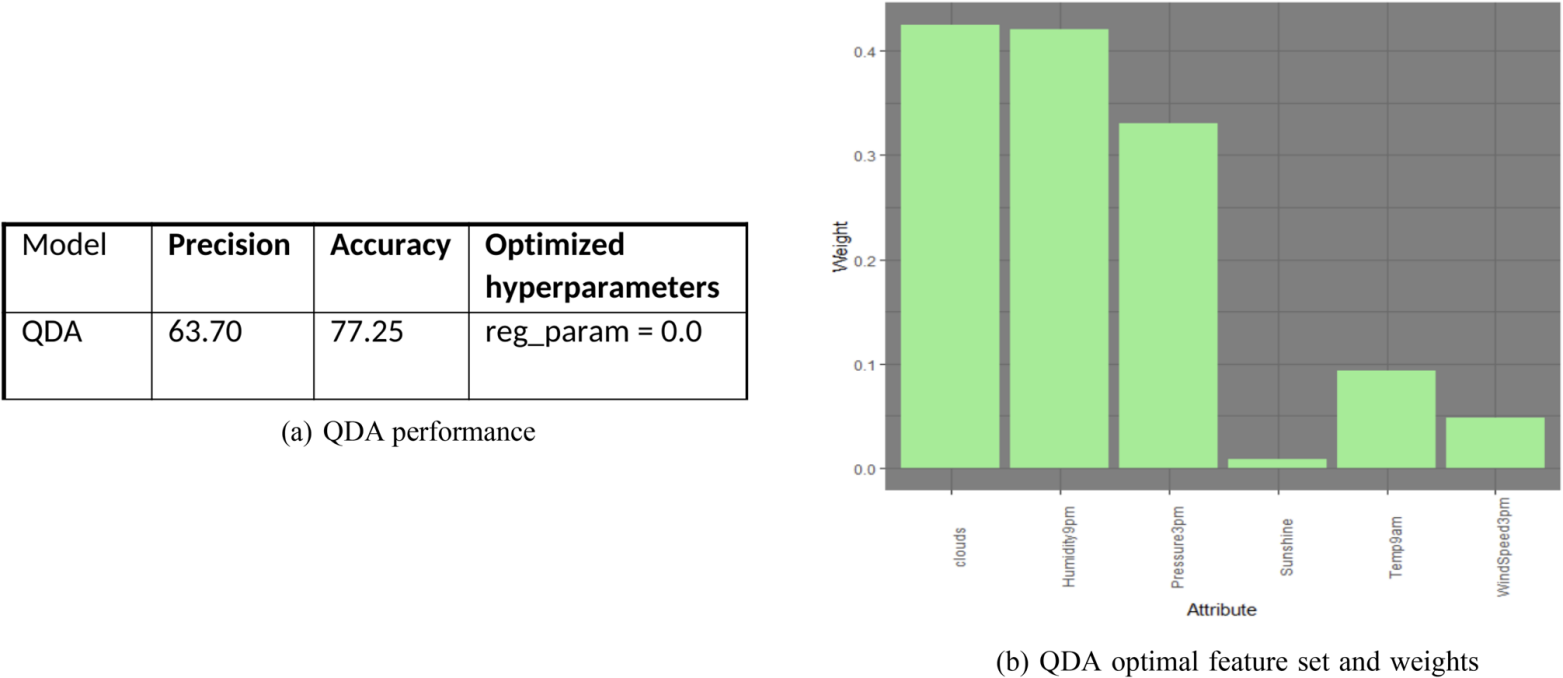
QDA performance and feature set.

Quadratic discriminant analysis selects the following features and weights and performs as demonstrated by the following Fig. 12a,b. Note that QDA model selects similar features to the LDA model, except flipping the morning features to afternoon features, and vice versa. Also, QDA model emphasized more on cloud coverage and humidity than the LDA model.

### K-Nearest neighbors

**Figure 13:**
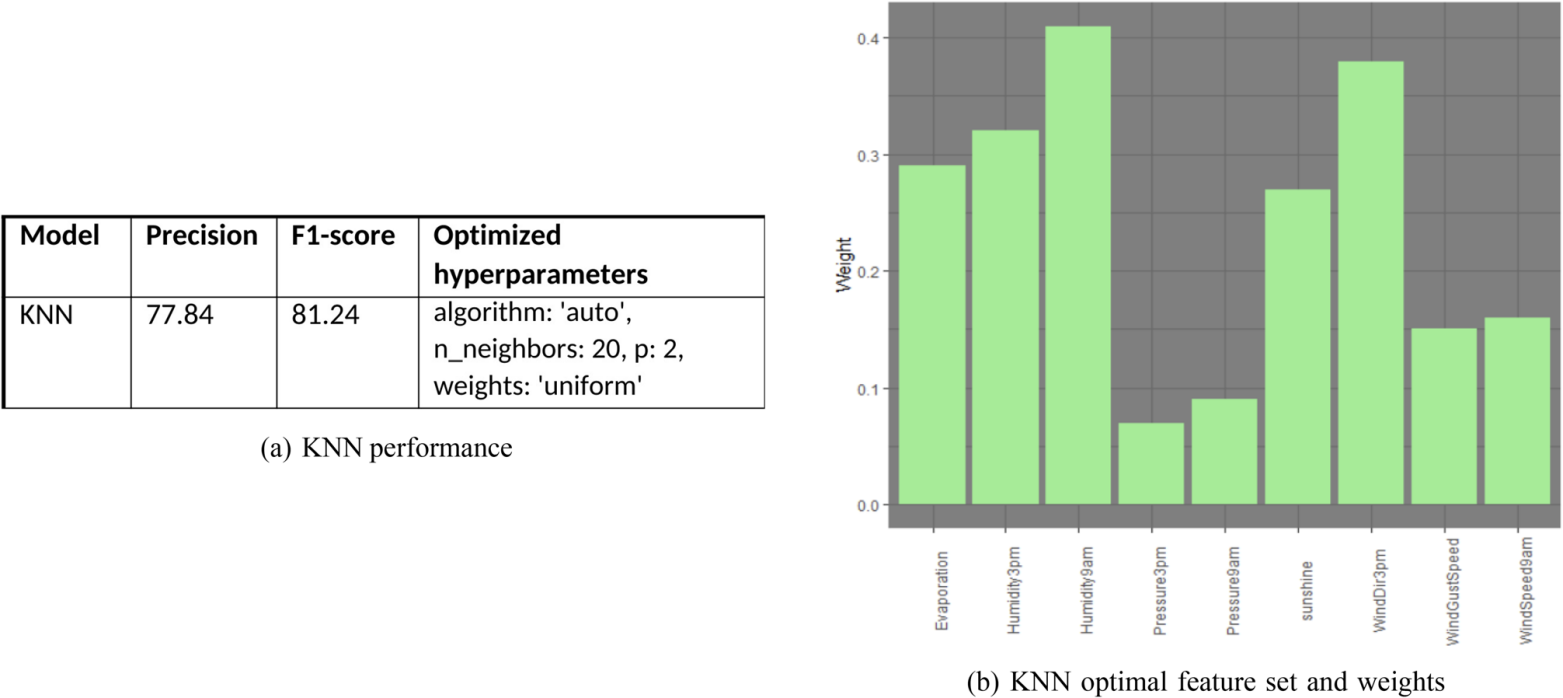
KNN performance and feature set.

From Fig. 13a, k = 20 is the optimal value that gives K-nearest neighbor method a better predicting precision than the LDA and QDA models. This may be attributed to the non-parametric nature of KNN. If the data is not linear or quadratic separable, it is expected that parametric models may show substandard performance. The performance of KNN classification is comparable to that of logistic regression.

The precision, f1-score and hyper-parameters of KNN are given in Fig. 13a. Also, Fig. 13b displays optimal feature set along with their feature weights.

### Decision tree

**Figure 14:**
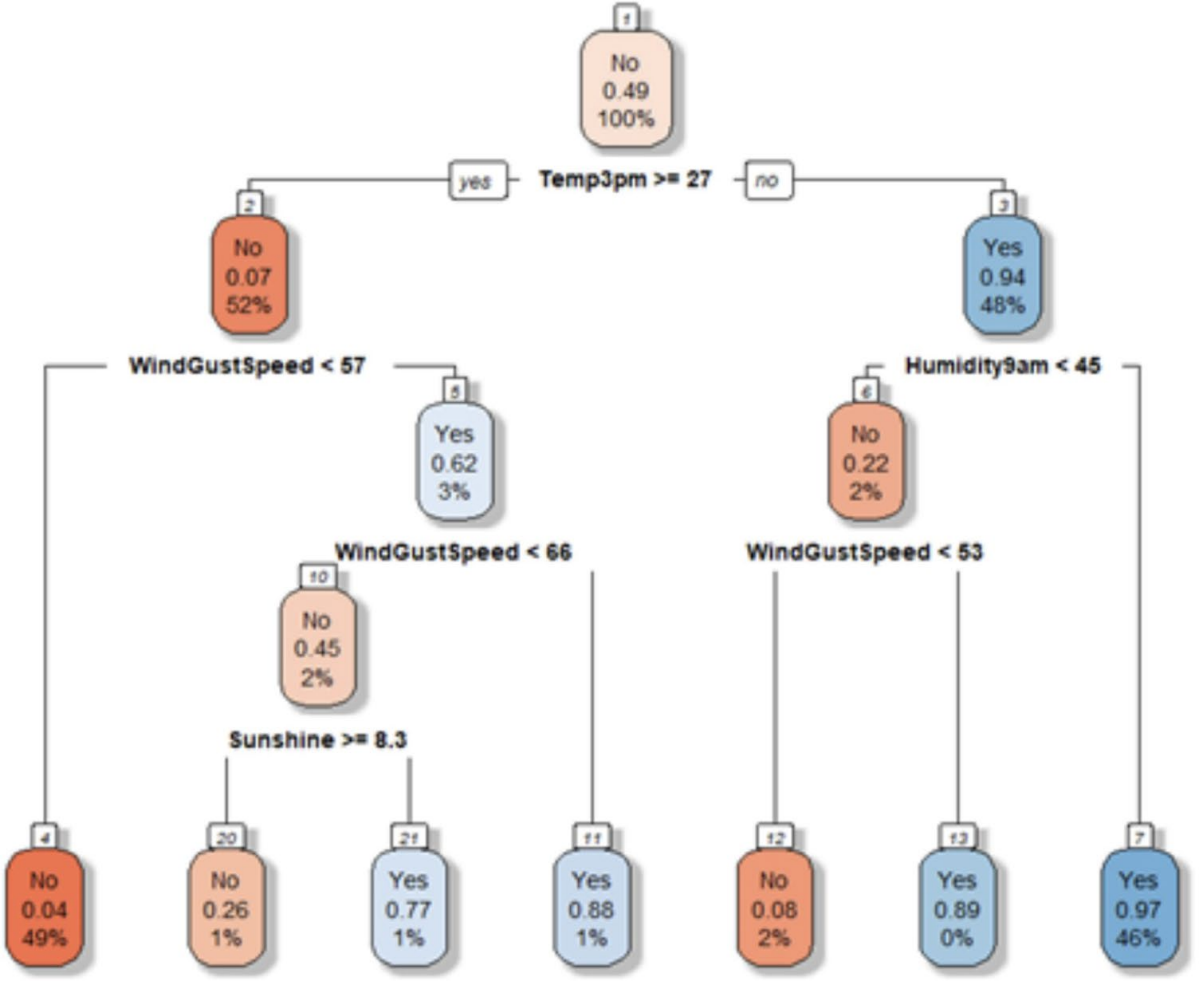
Decision tree.

The decision tree model was tested and analyzed with several feature sets. After generating the tree with an optimal feature set that maximized adjusted-R2, we pruned it down to the depth of 4. The decision tree with an optimal feature set of depth 4 is shown in Fig. 14.

**Figure 15:**
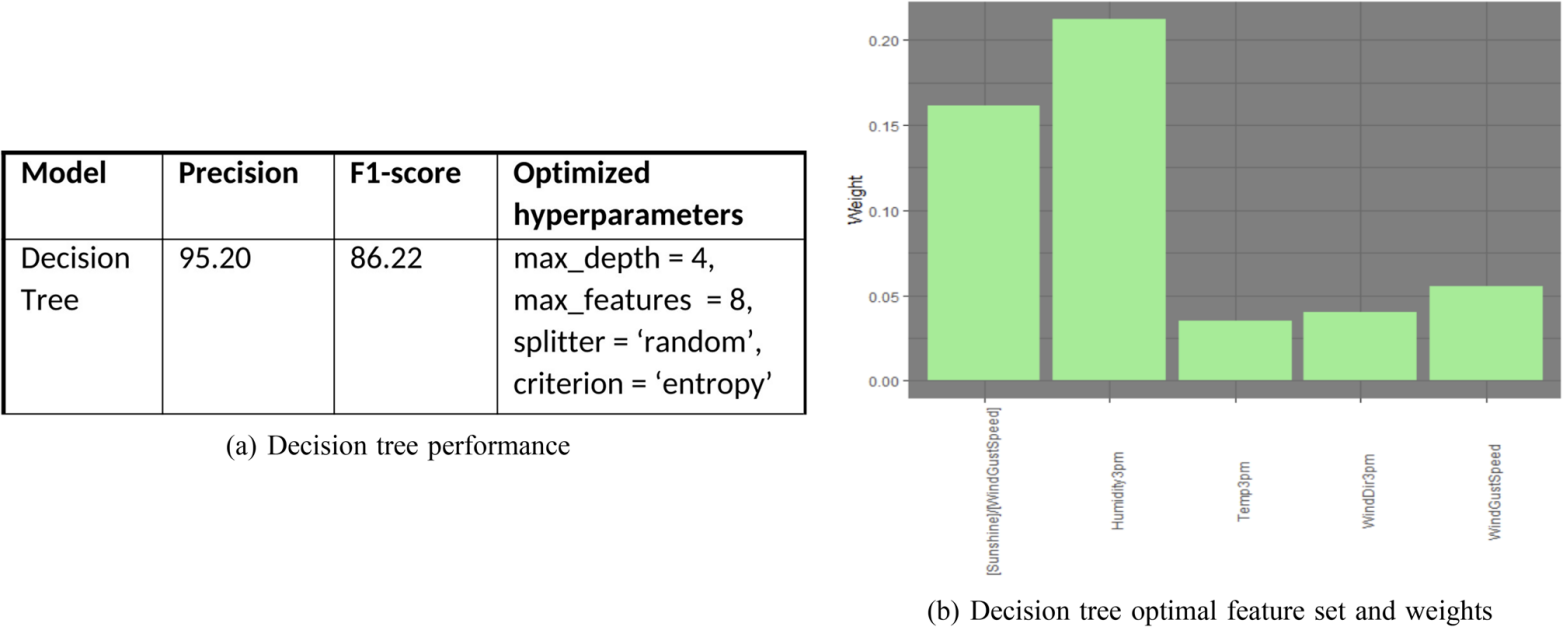
Decision tree performance and feature set.

Figure 15a displays the decision tree model performance. Also, Fig. 15b displays the optimal feature set with weights.

### Gradient boosting

**Figure 16:**
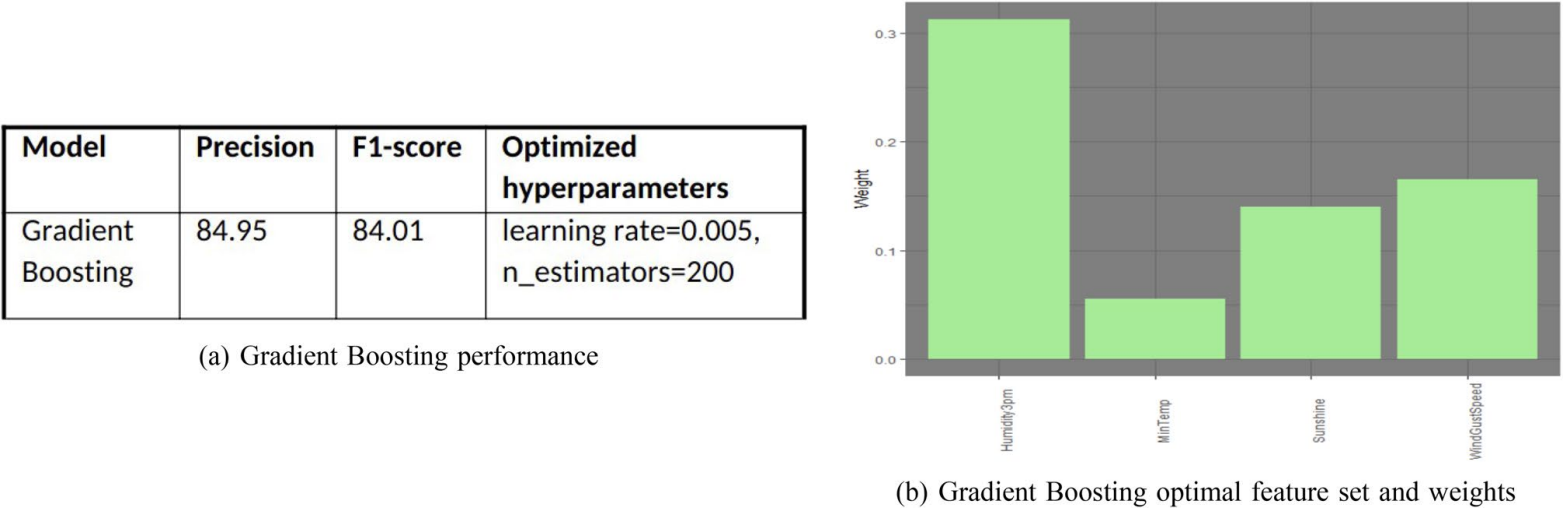
Gradient boosting performance and feature set.

We ran gradient boosted trees with the limit of five trees and pruned the trees down to five levels at most. The following are the associated features, their weights, and model performance. Note that gradient boosted trees are the first method that has assigned weight to the feature daily minimum temperature. Figure 16a displays the decision tree model performance. Also, Fig. 16b displays the optimal feature set with weights.

### Random forest

We perform similar feature engineering and selection with random forest model. Just like gradient forest model evaluation, we limit random forest to five trees and depth of five branches. Random forest model’s simple algebraic operations on existing features are noteworthy. Further, the model designated the following weights to the above features and demonstrated the following performance.

**Figure 17:**
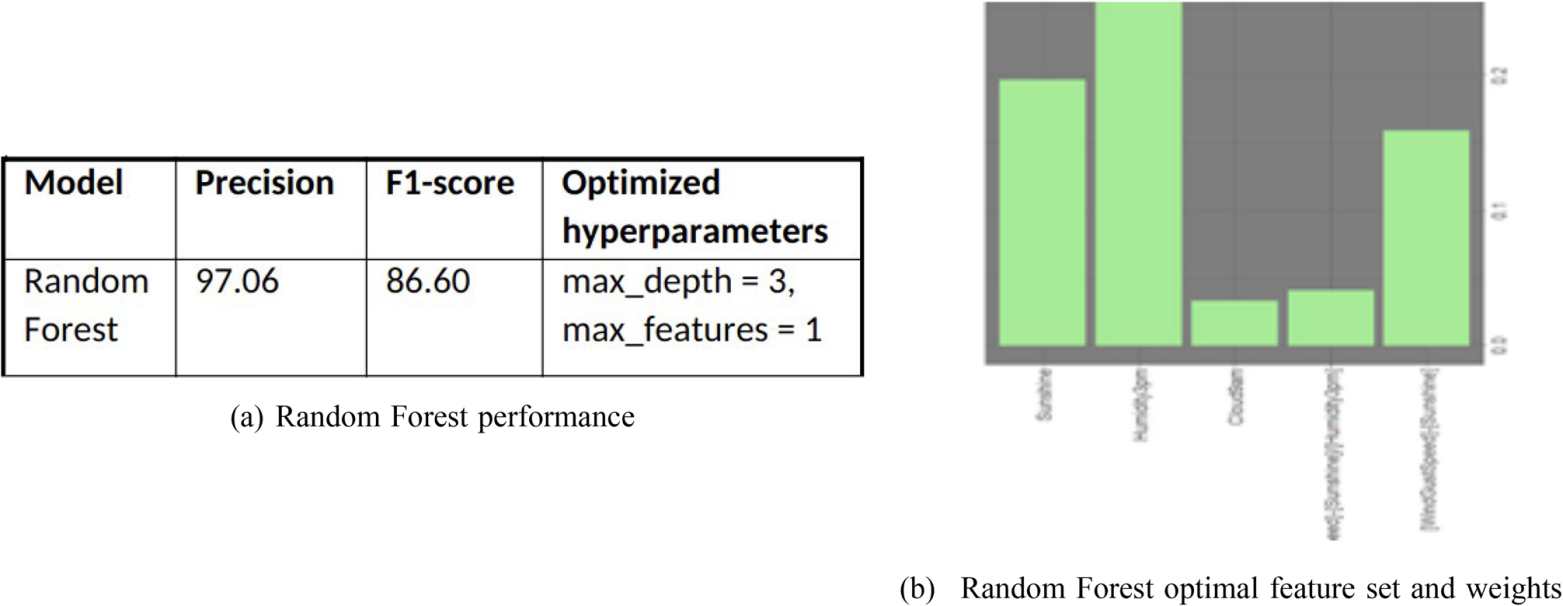
Random forest performance and feature set.

For the given dataset, random forest model took little longer run time but has a much-improved precision. This trade-off may be worth pursuing. Figure 17a displays the performance for the random forest model. Also, Fig. 17b displays the optimal feature set and weights for the model. It is noteworthy that the above tree-based models show considerable performance even with the limited depth of five or less branches, which are simpler to understand, program, and implement.

### Bernoulli Naïve Bayes

**Figure 18:**
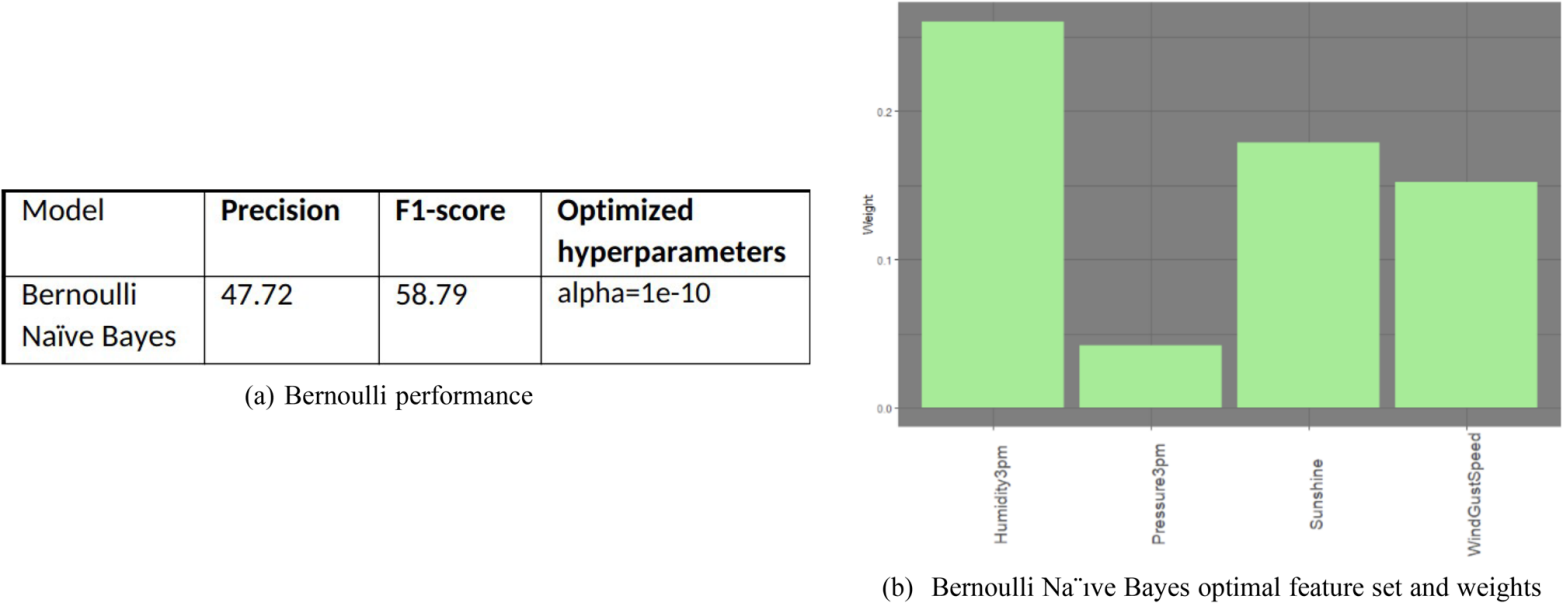
Bernoulli Naïve Bayes performance and feature set.

Figure 18a,b show the Bernoulli Naive Bayes model performance and optimal feature set respectively. It does not do well with much less precision. This could be attributed to the fact that the dataset is not balanced in terms of True positives and True negatives.

### Deep learning model

**Figure 19:**
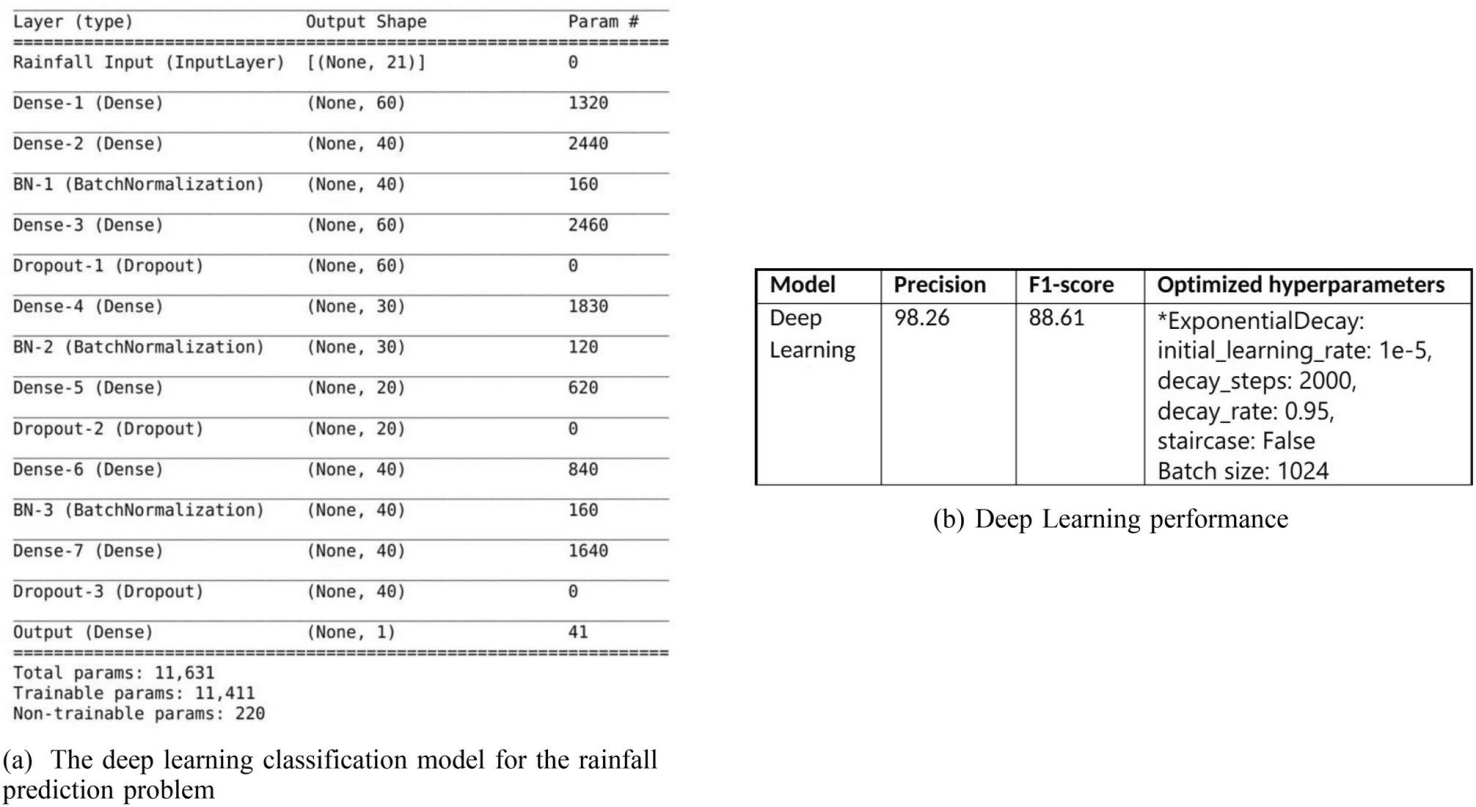
Deep learning model and performance.

To find out how deep learning models work on this rainfall prediction problem compared to the statistical models, we use a model shown in Fig. 19a. Although much simpler than other complicated models used in the image recognition problems, it outperforms all other statistical models that we experiment in the paper. The deep learning model for this task has 7 dense layers, 3 batch normalization layers and 3 dropout layers with 60% dropout.

**Figure 20:**
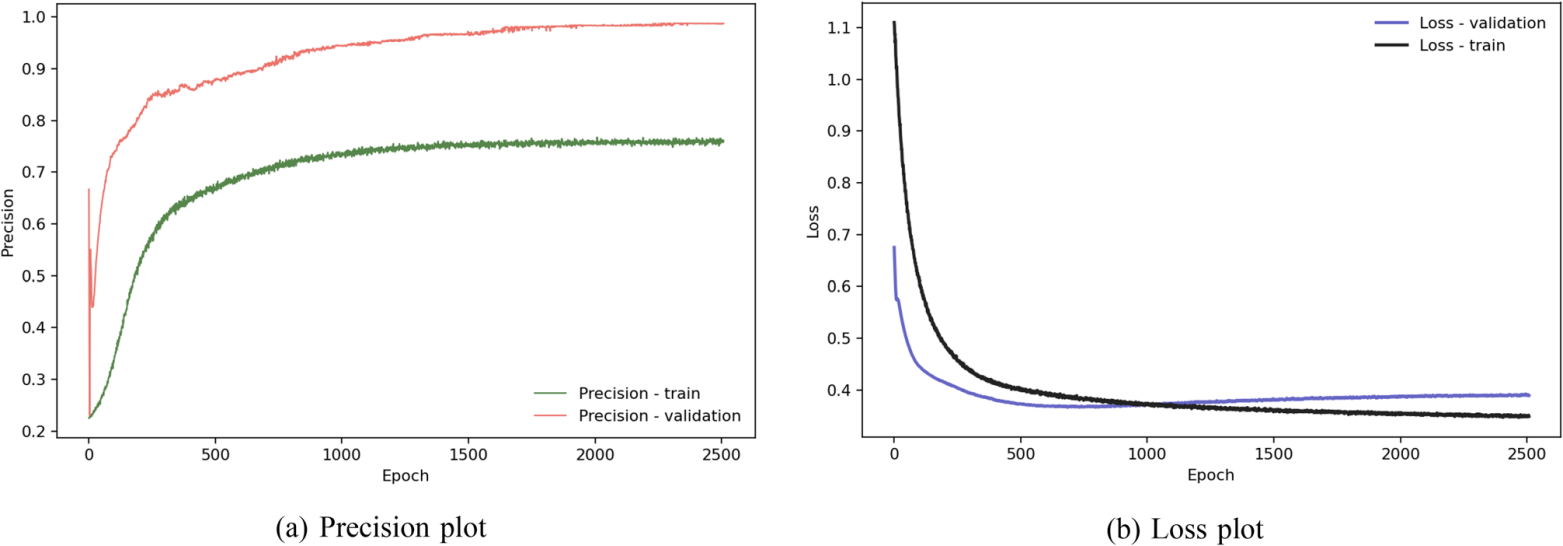
Deep learning model performance and plot.

Like other statistical models, we optimize this model by precision. Figure 20a shows the effect of the dropout layers onto the training and validation phases. During training, these layers remove more than half of the neurons of the layers to which they apply. Effectively they put a negative impact onto the model. In the validation phase, all neurons can play their roles and therefore improve the precision. Starting at epoch 2000, as shown in Fig. 20a,b, both precision and loss plots for validation do not improve any more. Figure 19b shows the deep learning model has better a performance than the best statistical model for this task—the logistic regression model, in both the precision and f1-score metrics.

## Conclusion

In this project, we obtained the dataset of 10 years of daily atmospheric features and rainfall and took on the task of rainfall prediction. We performed exploratory data analysis and generalized linear regression to find correlation within the feature-sets and explore the relationship between the feature sets. This enabled us to express correlated features into the form of one another. Moreover, we performed feature engineering and selected certain features for each of eight different classification models. We compared these models with two main performance criteria: precision and f1-score.

Based on the above performance results, the logistic regression model demonstrates the highest classification f1-score of 86.87% and precision of 97.14% within the group of statistical models, yet a simple deep-learning model outperforms all tested statistical models with a f1-score of 88.61% and a precision of 98.26%. This proves that deep learning models can effectively solve the problem of rainfall prediction.

## References

[1] Lim EP, Hendon HH, Boschat G, Hudson D, Thompson DWJ, Dowdy AJ, Arblaster JM (2019). Australian hot and dry extremes induced by weakening of the stratospheric polar vortex. Nat. Geosci..

[2] Sohn SJ, Kim WM (2020). Toward a better multi-model ensemble prediction of East Asian and Australasian precipitation during non-mature ENSO seasons. Sci. Rep..

[3] Sharmila S, Hendon HH (2020). Mechanisms of multiyear variations of Northern Australia wet-season rainfall. Sci. Rep..

[4] Munksgaard NC (2019). Data descriptor: Daily observations of stable isotope ratios of rainfall in the tropics. Sci. Rep..

[5] Benedetti-Cecchi L (2021). Complex networks of marine heatwaves reveal abrupt transitions in the global ocean. Sci. Rep..

[6] Ummenhofer CC, England MH, Mclntosh PC, Meyers GA, Pook MJ, Risbey JS, Gupta AS, Taschetto AS (2009). What causes southeast Australia’s worst droughts?. Geophys. Res. Lett..

[7] Xie SP, Deser C, Vecchi GA, Ma J, Teng H, Wittenberg AT (2010). Global warming pattern formation: Sea surface temperature and rainfall. J. Clim..

[8] Shi W, Wang M (2021). A biological Indian Ocean Dipole event in 2019. Sci. Rep..

[9] Brown BE, Dunne RP, Somerfield PJ, Edwards AJ, Simons WJF, Phongsuwan N, Putchim L, Anderson L, Naeije MC (2019). Long-term impacts of rising sea temperature and sea level on shallow water coral communities over a 40 year period. Sci. Rep..

[10] Darji, M. P., Dabhi, V. K., & Prajapati, H. B. Rainfall forecasting using neural network: A survey. In *Conference Proceeding—2015 International Conference on Advances in Computer Engineering and Applications, ICACEA 2015*. 10.1109/ICACEA.2015.7164782 (2015).

[11] Hu MJC, Root HE (1964). An adaptive data processing system for weather forecasting. J. Appl. Meteorol..

[12] Cook T, Folli M, Klinck J, Ford S, Miller J (1998). The relationship between increasing sea-surface temperature and the northward spread of *Perkinsus marinus* (Dermo) disease epizootics in oysters. Estuar. Coast. Shelf Sci..

[13] French MN, Krajewski WF, Cuykendall RR (1992). Rainfall forecasting in space and time using a neural network. J. Hydrol..

[14] Michaelides SC, Tymvios FS, Michaelidou T (2009). Spatial and temporal characteristics of the annual rainfall frequency distribution in Cyprus. Atmos. Res..

[15] Chauhan D, Thakur J (2014). Data mining techniques for weather prediction: A review. Int. J. Recent Innov. Trends Comput. Commun..

[16] Petre, E. G. A decision tree for weather prediction. *Seria Matematica˘-Informatica˘-Fizica˘*, Vol. 61, no. 1, 77–82 (2009).

[17] Sharif M, Burn DH (2006). Simulating climate change scenarios using an improved K-nearest neighbor model. J. Hydrol..

[18] Bureau of Meteorology, weather forecasts and radar, Australian Government. Accessed 26 Oct 2020. http://www.bom.gov.au/.

[19] Wei J, Chen H (2020). Determining the number of factors in approximate factor models by twice K-fold cross validation. Econ. Lett..

[20] McKenna S, Santoso A, Gupta AS, Taschetto AS, Cai W (2020). Indian Ocean Dipole in CMIP5 and CMIP6: Characteristics, biases, and links to ENSO. Sci. Rep..

[21] Li L, Wang YP, Beringer J, Shi H, Cleverly J, Cheng L, Eamus D, Huete A, Hutley L, Lu X, Piao S, Zhang L, Zhang Y, Yu Q (2017). Responses of LAI to rainfall explain contrasting sensitivities to carbon uptake between forest and non-forest ecosystems in Australia. Sci. Rep..

[22] Sheen KL, Smith DM, Dunstone NJ, Eade R, Rowell DP, Vellinga M (2017). Skilful prediction of Sahel summer rainfall on inter-annual and multi-year timescales. Nat. Commun..

[23] Dogan O, Taspınar S, Bera AK (2020). A Bayesian robust chi-squared test for testing simple hypotheses. J. Econ..

[24] Dutta R, Maity R (2018). Temporal evolution of hydroclimatic teleconnection and a time-varying model for long-lead prediction of Indian summer monsoon rainfall. Sci. Rep..

[25] Yaseen ZM, Ali M, Sharafati A, Al-Ansari N, Shahid S (2021). Forecasting standardized precipitation index using data intelligence models: regional investigation of Bangladesh. Sci. Rep..

[26] Praveen B, Talukdar S, Shahfahad Mahato S, Mondal J, Sharma P, Islam ARMT, Rahman A (2020). Analyzing trend and forecasting of rainfall changes in India using non-parametrical and machine learning approaches. Sci. Rep..

[27] Huang PW, Lin YF, Wu CR (2021). Impact of the southern annular mode on extreme changes in Indian rainfall during the early 1990s. Sci. Rep..

[28] Stone RC, Hammer GL, Marcussen T (1996). Prediction of global rainfall probabilities using phases of the Southern Oscillation Index. Nature.

