# Author Correction: Automated predictive analytics tool for rainfall forecasting

**DOI:** 10.1038/s41598-021-99054-w

**Published:** 2021-09-27

**Authors:** Maulin Raval, Pavithra Sivashanmugam, Vu Pham, Hardik Gohel, Ajeet Kaushik, Yun Wan

**Affiliations:** 1 Applied Artificial Intelligence Laboratory, University of Houston-Victoria, Victoria, USA; 2 NanoBioTech Laboratory, Florida Polytechnic University, Lakeland, USA

Correction to: *Scientific Reports* 10.1038/s41598-021-95735-8, published online 06 September 2021

The original version of this Article contained errors in the Affiliations.

Maulin Raval was incorrectly affiliated with ‘Department of Industrial Engineering, University of Houston, Victoria, USA’.

In addition, Pavithra Sivashanmugam, Vu Pham and Yun Wan were incorrectly affiliated with ‘Department of Computer Science, University of Houston-Victoria, Victoria, USA’.

The correct affiliations are listed below:

1. Applied Artificial Intelligence Laboratory, University of Houston-Victoria, Victoria, USA.

Maulin Raval, Pavithra Sivashanmugam, Vu Pham, Hardik Gohel & Yun Wan.

2. NanoBioTech Laboratory Florida Polytechnic University, Lakeland, USA.

Ajeet Kaushik.

The original Article has been corrected.

